# Critical assessment of different methods for quantitative measurement of metallodrug-protein associations

**DOI:** 10.1007/s00216-018-1328-8

**Published:** 2018-08-29

**Authors:** Luis Galvez, Sarah Theiner, Márkó Grabarics, Christian R. Kowol, Bernhard K. Keppler, Stephan Hann, Gunda Koellensperger

**Affiliations:** 1 Institute of Analytical Chemistry, Faculty of Chemistry, University of Vienna, Waehringer Strasse 38, 1090 Vienna, Austria; 2 Institute of Inorganic Chemistry, Faculty of Chemistry, University of Vienna, Waehringer Strasse 42, 1090 Vienna, Austria; 3 Department of Chemistry, Division of Analytical Chemistry, University of Natural Resources and Life Sciences - BOKU Vienna, Muthgasse 18, 1190 Vienna, Austria

**Keywords:** Metal-based anticancer drugs, High-throughput, ICP–MS, Elemental speciation analysis, Metal–protein interaction

## Abstract

Quantitative screening for potential drug–protein binding is an essential step in developing novel metal-based anticancer drugs. ICP–MS approaches are at the core of this task; however, many applications lack in the capability of large-scale high-throughput screenings and proper validation. In this work, we critically discuss the analytical figures of merit and the potential method-based quantitative differences applying four different ICP–MS strategies to ex vivo drug–serum incubations. Two candidate drugs, more specifically, two Pt(IV) complexes with known differences of binding affinity towards serum proteins were selected. The study integrated centrifugal ultrafiltration followed by flow injection analysis, turbulent flow chromatography (TFC), and size exclusion chromatography (SEC), all combined with inductively coupled plasma-mass spectrometry (ICP–MS). As a novelty, for the first time, UHPLC SEC-ICP–MS was implemented to enable rapid protein separation to be performed within a few minutes at > 90% column recovery for protein adducts and small molecules.

**Electronic supplementary material:**

The online version of this article (10.1007/s00216-018-1328-8) contains supplementary material, which is available to authorized users.

**Graphical abstract:**
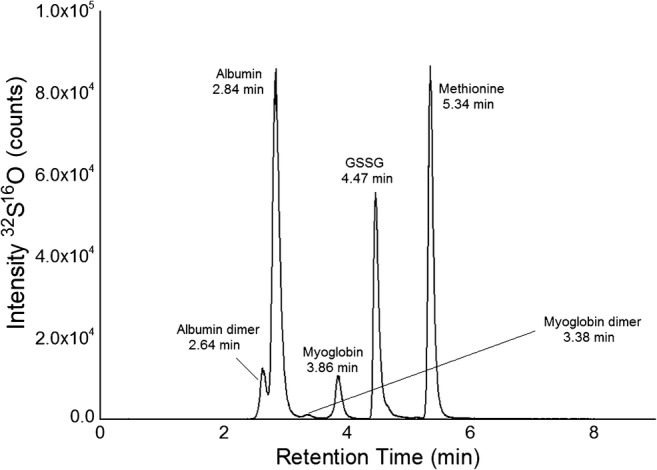
Quantitative screening for potential drug–protein binding is an essential step in developingnovel metal-based anticancer drugs

## Introduction

The development of metal-based anticancer drugs continues to be a topical research theme. In fact, a recent upsurge of activities regards the development of metal-based compounds and non-classical platinum complexes whose mechanism of action is distinct from known drugs such as the clinically established cisplatin [1–3]. Many metal-based compounds have been synthesized by redesigning the existing chemical structure through ligand substitution or building the entire new compound with enhanced safety and cytotoxic profile [3, 4].

As in any drug development, studying the chemistry of the drug in solution-based ex vivo experiments is a first crucial step. In the case of metal-based anticancer drugs, in vitro protein binding studies received major attention, as it was recognized soon that protein binding occurred for many compounds already in short time scales in human blood and would be therefore decisive regarding drug delivery, drug deactivation, or vice versa drug activation. For example, whereas for platinum (II)-based drugs such as cisplatin binding to serum proteins, especially human serum albumin (HSA) leads to deactivation and lower bioavailability of the drug [5], other concepts exploit HSA for targeted drug delivery to the tumor tissue [6, 7]. HSA is known to accumulate in cancer cells, an advantage that was investigated by the preparation of functionalized platinum (IV) prodrugs that were designed to bind selectively to HSA [8–10].

Evidently, tools of trade for this type of ex vivo investigations are offered by elemental speciation approaches utilizing ICP–MS as a detection technique of choice [11–14]. ICP–MS featuring species unspecific quantification capabilities was combined with intact-protein separation- or fractionation techniques ensuring native, i.e., mild chemical conditions, being the latter a prerequisite ensuring that protein binding was not reversed upon chromatographic separation, as drug protein association could be based on coordinative and/or covalent binding [3, 11, 15–19]. A comprehensive review about the different approaches is given elsewhere [20, 21]. In brief, capillary electrophoresis (CE) and SEC have been the most commonly applied native separation methods [22, 23]. Already in 1999 [24], SEC combined with ICP–MS emerged as essential tool for studying the interaction of metallodrugs and serum proteins and has seen numerous applications ever since in support of new drug design concepts. Despite this success, the separation was considered a low-resolution, time-consuming method. The involved length of single experiments was regarded as major disadvantage especially when kinetics studies were addressed. Typical chromatographic separation times ranged between 20 and 30 min [25]. As an alternative to on-line separation, several studies resorted to an off-line fractionation technique, which had been developed in the late 1960s [26], namely centrifugal ultrafiltration using cut-off filters. As a key advantage, this strategy is technically very simple; however, it became very clear soon that filter material selection and preconditioning strategy was crucial regarding drug recovery [27–30], making this technique more tedious than expected. Flow injection (FI)-ICP–MS [28, 31] proved to be a valuable method addressing the metal-based drug distribution between low (LMF) and high molar mass fraction (HMF) in very small sample volumes. In some cases, off-line protein removal was followed by LC-ICP–MS analysis in order to assess potential low molar mass transformation products of the metal-based drugs [16, 27, 32]. Only recently, on-line protein removal by turbulent flow chromatography (TFC) was introduced in combination with ICP–MS for studying metal-based anticancer drugs and their protein binding. TFC was first developed in the late 1990s as an emerging, alternative approach to study biological samples by extracting on-line the HMF and analyzing the LMF. In TFC, very high mobile phase linear velocities are combined with stationary phases consisting of large porous particles (30–80 μm). This combination leads to a turbulent flow regime which generates a mass transfer cut-off allowing large molecules, e.g., proteins, to pass without any interaction. Small molecules can interact with the functional groups of the stationary phase and are then eluted subsequently by an appropriate solvent or buffer [33–36].

The aim of this work, thus, was to provide a comparison between different state-of-the-art methods focusing on the aspect of quantitative protein binding studies. The investigated approaches offered a varying degree of automation. The objective was to shed light on crucial and sometimes overlooked shortcomings that make a generalizable straightforward quantitative application without considering the chemistry of the metallodrugs difficult. Moreover, as a novelty, UHPLC SEC was combined with ICP–MS detection for studying metallodrug–protein interaction in human serum. Various approaches have been developed to improve the speed of size-exclusion chromatography [37], with the recently introduced UHPLC SEC or sub-2 μm technique being one of the most promising ones, reducing both eddy dispersion and resistance towards mass transfer, two of the most important factors contributing to band broadening in liquid chromatography [38]. For SEC, these sub-2 μm materials have been introduced several years ago; however, their application in speciation studies is novel. A recent review reports on developments in UHPLC SEC [39] including the key application areas such as, e.g., purity monitoring of protein-related compounds in industry, the development process of biotherapeutic proteins, the field of proteomics, and the polymer industry [40, 41]. To the best of our knowledge, this technology has not been applied to speciation analysis with ICP–MS detection so far.

Despite the fact that numerous methods addressing metallodrug-biomolecule interactions are available, a comparative study on different metallodrugs (different ligand chemistry) is still lacking. Hence, next to size-exclusion-based approaches, the cross-validation study involved centrifugal filtration and turbulent flow chromatography, always in combination with ICP–MS detection. All investigated approaches were applied to test compounds in serum incubations. For this purpose, two platinum (IV)-based prodrugs (1) with selective albumin-binding properties by a maleimide moiety and (2) a negative control being non-reactive towards biomolecules with a succinimide moiety were selected. The novel drugs release oxaliplatin upon reductive activation. The compound with selective albumin binding was already tested in vivo colon carcinoma-bearing mice and treatment resulted in significantly reduced tumor growth and even disease stabilization. Additionally, for a very similar compound, a highly increased plasma half-life and very efficient tumor accumulation were observed [8].

## Experimental

### Reagents, chemicals, and standards

**Fig. 1:**
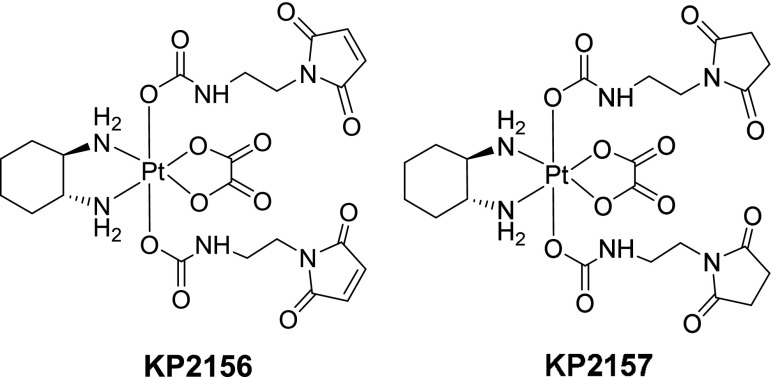
Chemical structures of KP2156 containing a maleimide moiety designed to bind covalently to the cystein residues of serum proteins via maleimide-thiol coupling reaction and KP2157 containing a succinimide moiety lacking of the ability to bind to sulfhydryl groups [8]

A reagent I grade water (> 10 MΩ cm^−1^ resistance according to ISO 3696 water specifications) purification system (Ultra Clear basic Reinstwassersystem, SG Wasseraufbereitung und Regenerierstation GmbH, Barsbüttel, Germany) was used to obtain purified water. Ammonium acetate (≥ 99%), glutathione disulfide (≥ 98%), albumin from human serum (96–99%), and myoglobine from equine skeletal muscle (95–100%) were purchased from Sigma Aldrich, St. Louis, MO, USA. Methionine (≥ 99%) was purchased from Merck (Darmstadt, Germany). Pt standard (1002 ± 6 μg/mL) was purchased from Inorganic Ventures, Christiansburg, VA, USA. Fetal calf serum (FCS) was kindly provided by Prof. Walter Berger from the Institute of Cancer Research, University of Vienna, Vienna, Austria. The analytical figures of merit for metallodrug studies were assessed by the use of two platinum (IV) compounds synthesized according to literature procedures [8] at the Institute of Inorganic Chemistry, University of Vienna, Vienna, Austria. The structures of these compounds, KP2156 and KP2157, are shown in Fig. 1. More specifically, the selection comprised KP2156 (trans-bis(2-maleimideethylcarbamato)-dihydroxido(1R,2R-diamminocyclohexane)-oxalatoplatinum (IV)), a maleimide-functionalized Pt (IV) complex with oxaliplatin core and KP2157 (trans-bis(2-succinimideethylcarbamato)-dihydroxido(1R,2R-diamminocyclohexane)-oxalatoplatinum (IV)), its succinimide analog [8]. While KP2156 is designed to bind covalently to the cystein residues of serum proteins via maleimide-thiol coupling reaction, in order to utilize the enhanced permeability and retention effect for targeting, its succinimide derivative, KP2157, inherently lacks the ability to form covalent bounds with sulfhydryl groups, due to the missing double bonds in the axial linker moiety (Fig. 1). Prior kinetics studies by UHPLC SEC in our laboratory showed this difference in the affinity towards serum proteins (see Electronic Supplementary Material (ESM) Table S1 for chromatography and ICP–MS conditions and Fig. S1 for chromatograms).

### Ex vivo incubations

Stock solutions of KP2156 and KP2157 were prepared in water (1 μM) right before the incubation in FCS. 100 μL of the stock solution was incubated in 900 μL of FCS (corresponding to a molar ratio of drug:albumin of 1:6000 approximately) for 45 min at 37 °C. Immediately prior to ICP–MS determination, the incubations were diluted by a factor of 10 resulting in a drug concentration of 0.01 μM. In all experiments, aqueous solutions of the drugs were employed, as the drugs are stable under these conditions [8].

### ICP–MS

The ICP–MS instrument used for the quantification studies with conventional SEC and TFC was an iCAP-QMS Thermo Scientific (Bremen, Germany) with oxygen (purity 5.0, Linde Gas GmbH, Vienna, Austria) as reaction gas and Qtegra Intelligent Scientific Data Solution (version 2.4.1800.33) for the data treatment. For the quantification in the centrifugal ultrafiltration studies and for UHPLC SEC, the Agilent 8800 ICP–MS/MS (Agilent Technologies, Tokyo, Japan) was used with Agilent MassHunter software package (Work- station Software, Version C.01.03, 2016) for the data treatment. The ICP–MS parameters are summarized in Table 1.

**Table 1:** ICP–MS operation parameters

Instrumentation ICP–MS	iCAP-QMS Thermo Scientific	ICP–MS/MS Agilent 8800
Nebulizer	PFA-ST	MicroMist
Spray chamber	Cyclonic	Scott double-pass
Nebulizer gas flow	1.01 L/min	1.05 L/min
Aux. gas	0.99 L/min	0.90 L/min
Plasma gas	14 L/min	15 L/min
Reaction gas	0.370 mL/min	0.300 mL/min
ICP RF Power	1550 W	1550 W
m/z measured	194.97, 47.97	195, 48

### SEC and UHPLC SEC

The Agilent 1260 infinity Bio-inert HPLC system (Agilent Technologies, Waldbronn, Germany) was employed for the UHPLC SEC measurements. For the quantification studies with conventional SEC, the Thermo Scientific Transcend HPLC system (San Jose, CA, USA) was used. The chromatographic conditions are given in Table 2.

**Table 2:** Chromatographic conditions

Instrumentation HPLC	Agilent 1260 infinity Bio-inert	Thermo Scientific Transcend system
HPLC Column	Acquity UPLC Protein BEH SEC, 4.6 × 150 mm, 125A, 1.7 μm, 1 kDa–80 kDa, Waters	BioBasic SEC-60 A, 4.6 × 250 mm, 5 μm, 0.1 kDa–10,000 kDa Thermo
Eluent	50 mM CH_3_COONH_4_, pH = 6.0	50 mM CH_3_COONH_4_, pH = 6.0
Flow rate	400 μL/min	250 μL/min
Injection volume	5 μL	10 μL
Column temperature	30 °C	Room temperature
Autosampler Temperature	4 °C	4 °C
Combined with	ICP–MS/MS Agilent 8800	ICP-QMS iCAP Thermo Scientific

### Centrifugal ultrafiltration

Centrifugal ultrafiltration was carried out using Amicon Ultra 0.5-mL centrifugal regenerated cellulose membrane filters, with a 10-kDa molecular weight cut-off (MWCO) (Millipore Co, Carrigtwohill, Co. Cork, Ireland). The samples were ultracentrifuged with a Hermle Z 466 K device with a precooled rotor (4 °C) at a G-force of 12,500*g*. For preconditioning of the membrane filters and ultracentrifugation of the drug solutions, a centrifugation time of 60 min and 15 min was used, respectively. The filters used for centrifugal ultrafiltration were preconditioned using the solvents with the highest recovery for the drugs, respectively. For KP2156, 50 mM CH_3_COONH_4_, pH = 6.0 was employed, whereas for KP2157 FCS was used. Flow injection measurements for platinum quantification were performed using an Agilent 1260 Infinity Bio-inert HPLC system (Agilent Technologies, Waldbronn, Germany) coupled to an Agilent 8800 ICP–MS/MS (Agilent Technologies, Tokyo, Japan). The HPLC system was operated with CH_3_COONH_4_ (50 mM, pH = 6) as eluent, a flow rate of 250 μL/min, and an injection volume of 10 μL. The ICP–MS operation parameters are summarized in Table 1.

### TFC

**Fig. 2:**
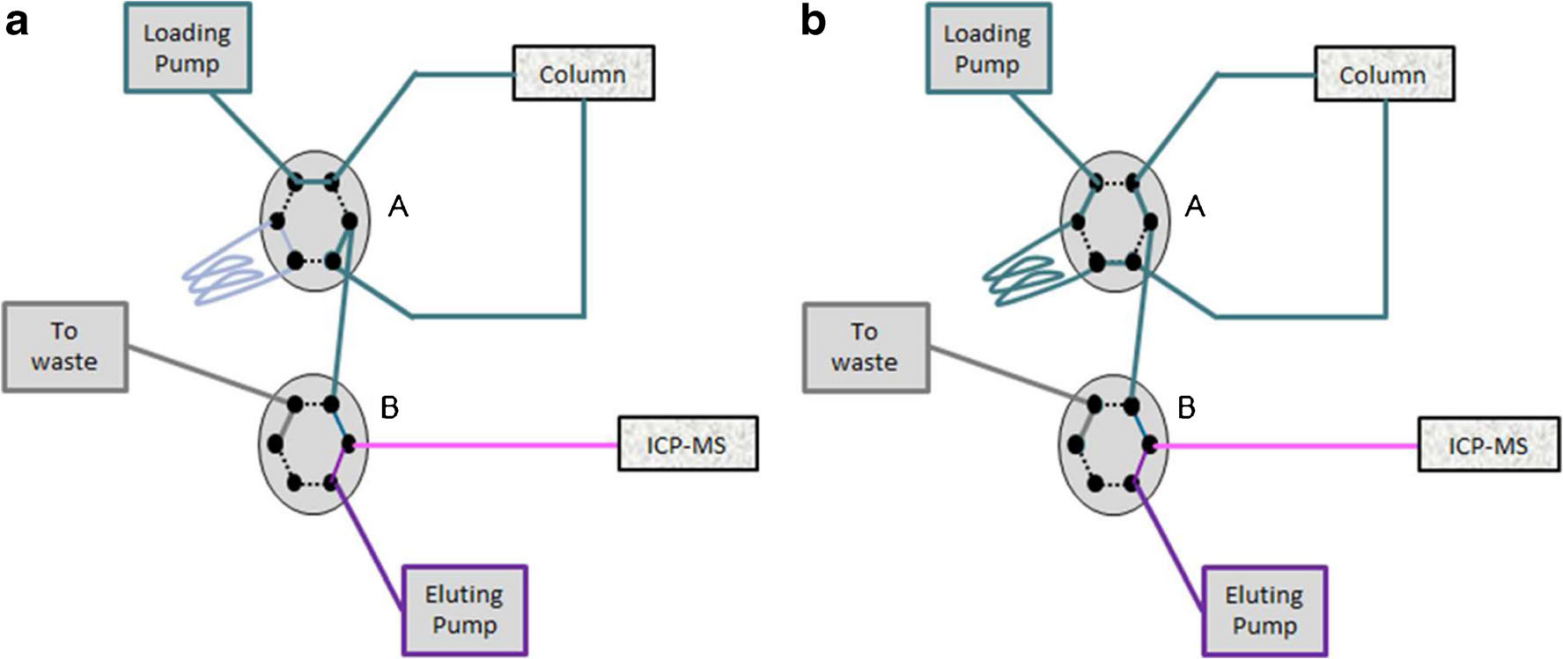
**A** At turbulent flow conditions (1 mL/min, 20% MeOH, delivered by the loading pump, while the eluting pump is set to 0 mL/min flow), the HMF (bound fraction of metallodrug) shows no retention on the column and can hence be directed to the ICP–MS (depending on the valve B position). The free drug corresponding to the low molar mass fraction LMF is retained. Subsequently, the flow delivered by the loading pump is reduced to 0.2 mL/min (20% MeOH) and mixed with the flow delivered by the eluting pump (0.8 mL/min 100% buffer). In the following step, the valve A is switched (**B**) and the “loading pump”- eluent, mixed with a solvent plug of 100% MeOH in a loop, is sent back-flush to the column. This high organic solvent conditions (0.2 mL/min) result in elution of the LMF from the column. Before reaching the ICP–MS, the latter flow is combined with the flow of the eluting pump, in order to reduce the MeOH content. Throughout all steps, the flow rate entering the introduction system of the ICP–MS is set to 1 mL/min. Finally, the last steps involve column cleaning and the loop loading with 100% MeOH. In the last step, the valve A is switched to the initial position (**A**) and the column is preconditioned for the next injection

TFC combines SEC and traditional stationary phase chemistry. In this case, a reversed phase was employed. The HPLC used was a HPLC Thermo Scientific Transcend system (San Jose, CA, USA) coupled to an ICP-QMS iCAP Thermo Scientific (Bremen, Germany). The separation and elution of the HMF and LMF were carried out by a two inert six-port valves present in this HPLC instrument (Fig. 2). The chromatographic conditions and the method are shown in Table 3.

**Table 3:** Chromatographic conditions and TFC method

Chromatographic conditions																
HPLC Column								Fluoro XL 0.5 × 50 mm								
Eluent								Eluent B 50 mM CH_3_COONH_4_, pH = 6.0, Eluent C MeOH								
Injection volume								10 μL								
Column temperature								Room temperature								
Autosampler temperature								4 °C								
TFC Method																
Step	Start	Sec	Loading pump						Tee	Loop	Eluting pump					
			Flow	Grad	A	B	C	D			Flow	Grad	A	B	C	D
1	0	40	1	Step	–	80	20	–	T	out	0	step	–	80	20	–
2	0.67	5	0.2	Step	–	80	20	–	T	out	0.8	step	–	100	–	–
3	0.75	55	0.2	Step	–	80	20	–	T	in	0.8	step	–	100	–	–
4	1.67	10	1	Step	–	–	100	–	===	in	1	step	–	80	20	–
5	1.83	200	1	Step	–	–	100	–	===	in	1	step	–	80	20	–
6	5.17	240	1	Step	–	80	20	–	===	out	1	step	–	80	20	–

## Results and discussions

### SEC and UHPLC SEC

**Fig. 3:**
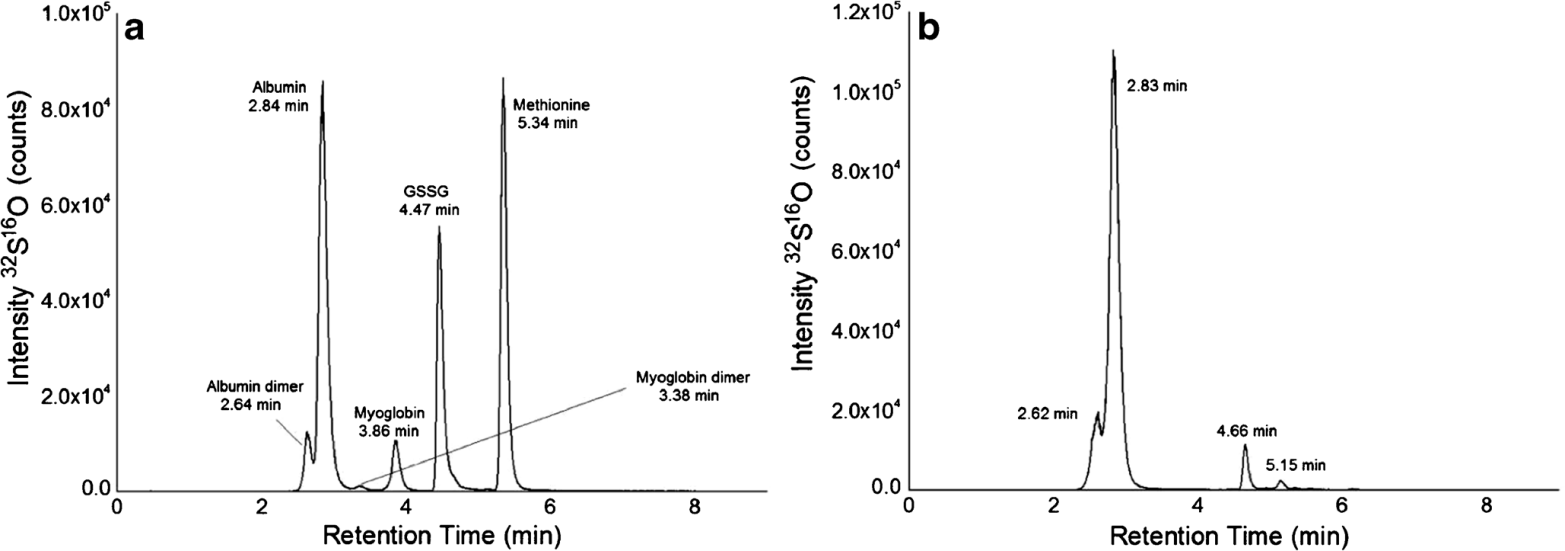
UHPLC SEC chromatograms with the column Acquity UPLC Protein BEH SEC, 4.6 × 150 mm, 125A, 1.7 μm, size range 1 kDa–80 kDa, at a flow rate of 400 μL/min and an injection volume of 5 μL of (**A**) a protein/amino acid mixture containing standards of HSA (66 kDa)(~ 9.32 μM), Myoglobin (17 kDa) (~ 34.6 μM), glutathione disulfide (GSSG, 0.6 kDa) (~ 375 μM) and methionine (0.1 kDa) (~ 1700 μM) and (**B**) undiluted FCS. Sulfur was monitored as ^32^S^16^O at m/z 47.97 with oxygen as reaction gas

The UHPLC SEC-ICP–MS approach established in this work relied on the most advanced columns available on the market. More specifically, the implemented SEC column was based on the use of high pore-volume ethylene-bridged hybrids (BEH) particles with diol-bonded surface which combines an increase of ~ 75% in pore volume with the required mechanical rigidity to maintain their integrity under high pressure and shear conditions. This stationary phase shows significantly reduced acidity of residual silanol groups, thereby reducing the contribution of unwanted ionic interaction between separated molecules and the stationary phase. UHPLC SEC when compared to conventional SEC for methionine (see ESM Table S2), (compound selection based on comparable retention time factors on the two columns) showed significantly higher separation efficiency. In fact, the number of theoretical plates/m was increased drastically, due to smaller peak widths. Figure 3A, B shows typical UHPLC SEC-ICP–MS separations accomplished within a few minutes. The size ladder consisting of a protein/amino acid mixture (Fig. 3A) and undiluted FCS (Fig. 3B) was analyzed monitoring sulfur (m/z 47.97 with oxygen as reaction gas). The retention time of the most abundant protein in FCS corresponded to albumin (2.83 min). The optimal size range of the used column was 1–80 kDa, presenting thus, the dimer of albumin (132 kDa) in the exclusion limit/void volume (2.64 min).

The analytical figures of merit for metallodrug studies were assessed by the use of KP2156 and KP2157 which served as perfect structural analogs of each other, with significantly different affinity towards serum proteins as explained in the experimental part and shown in the ESM (Table S1 and Fig. S1).

As can be readily seen in Table 4, UHPLC SEC-ICP–MS resulted in excellent recoveries for Pt. Regardless whether drug serum incubations (45 min of incubation of KP2156 and KP2157 in FCS at 37 °C, resulting in a molar ratio of drug:albumin of ~ 1:6000) or drug only (0.01 μM) was analyzed, the mass balance for Pt was complete as the diol bonded surface of the porous hybrid organic/inorganic particles featured significantly decreased acidity of the residual silanol groups. In this way, the SEC eluent composition could be optimized with the aim of reducing salt content thereby increasing the robustness of the method without compromising protein or small molecule recovery. Moreover, excellent limits of detection could be obtained which were comparable to conventional SEC (Table 7), despite the fact that the latter approach utilized lower flow rates (by a factor of 2) and higher injection volumes (factor of 2). Excellent intermediate repeatabilities of 2% were observed for UHPLC SEC-ICP–MS (flow rate of 400 μL/min and an injection volume of 5 μL) upon *n* = 18 and *n* = 15 injections over 24 h for albumin (monitored via sulfur) and a serum incubation of KP2156 (monitored via Pt). The chromatographic retention time repeatability ranged at 0.4 and 0.5% respectively.

**Table 4:** Analytical figures of merit of the standards and the samples

Acquity UPLC Protein BEH SEC, 4.6 × 150 mm, 125A, 1.7 μm				
Compound	Recovery (%)	LOD (nM) 3σ criterion	LOD (μg/L Pt) 3σ criterion	Absolute LOD (fg Pt) 3σ criterion
KP2156	100 ± 5	0.50	0.10	500
KP2157	99 ± 9	0.51	0.10	500
KP2156 in serum	101 ± 4	0.71	0.14	700
KP2157 in serum	97 ± 3	0.36	0.07	350

### Centrifugal ultrafiltration

An orthogonal approach to SEC and UHPLC SEC-ICP–MS suitable to ex vivo screening of metallodrug-protein interaction is offered by centrifugal ultrafiltration. The sample is fractionated into a protein rich high molar mass fraction (HMF) and a filtrate which constitutes the low molar mass fraction (LMF). The established procedure is to analyze the total Pt content of the sample prior to fractionation, followed by the determination of the Pt content of the filtrate. Due to practical reasons, the HMF is derived from the difference of the two obtained Pt amounts provided that the mass balance of the procedure is complete. Although it could seem a straightforward method, the often encountered poor recoveries of the metal-based drugs pose sever limitations to this otherwise simple method. As the quantitative results critically depend on filter materials [27–30] and preconditioning, we evaluated different preconditioning strategies for the two test compounds KP2156 and KP2157 (Table 5). Regenerated cellulose filters with a cut-off limit of 10 kDa were selected as these filters showed excellent recoveries for different Pt(II) compounds [27]. In this study, regardless which preconditioning method was tested, the recovery of the free drug from diluted drug standards was poor. However, the obtained LODs were lower by a factor of 10 compared to the SEC strategy. The LODs ranged at 0.03 nM (0.006 μg/L of Pt) and 0.01 nM (0.002 μg/L of Pt) for KP2156 and KP2157, respectively (3σ criterion).

**Table 5:** Centrifugal ultrafiltration

Standards (0.1 μM)		
Compound	Preconditioning	Recovery (%)
KP2156	–	8.7 ± 1.1 *n* = 3
KP2156	Milli-Q water	10 ± 1 *n* = 3
KP2156	50 mM CH_3_COONH_4_, pH = 6.0	9.6 ± 2.1 *n* = 3
KP2156	FCS	7.5 ± 1.4 *n* = 3
KP2157	–	38 ± 2 *n* = 9
KP2157	Milli-Q water	41 ± 3 *n* = 9
KP2157	50 mM CH_3_COONH_4_, pH = 6.0	38 ± 6 *n* = 9
KP2157	FCS	60 ± 3 *n* = 9

### TFC-ICP–MS

**Fig. 4:**
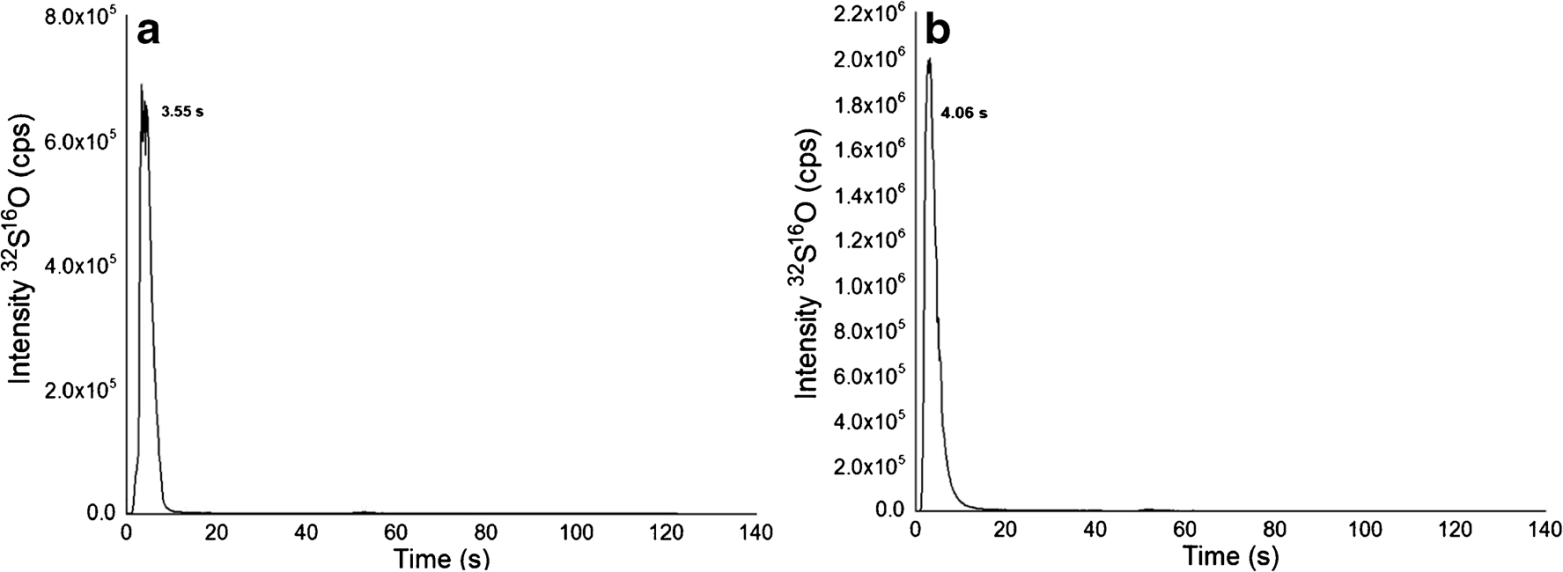
TFC chromatograms of (**A**) a standard of albumin 3.79 nM. The LOD was 0.006 nM (3σ criterion). **B** FCS diluted 1:10 in water. Sulfur was monitored at m/z 48 with oxygen as reaction gas

**Fig. 5:**
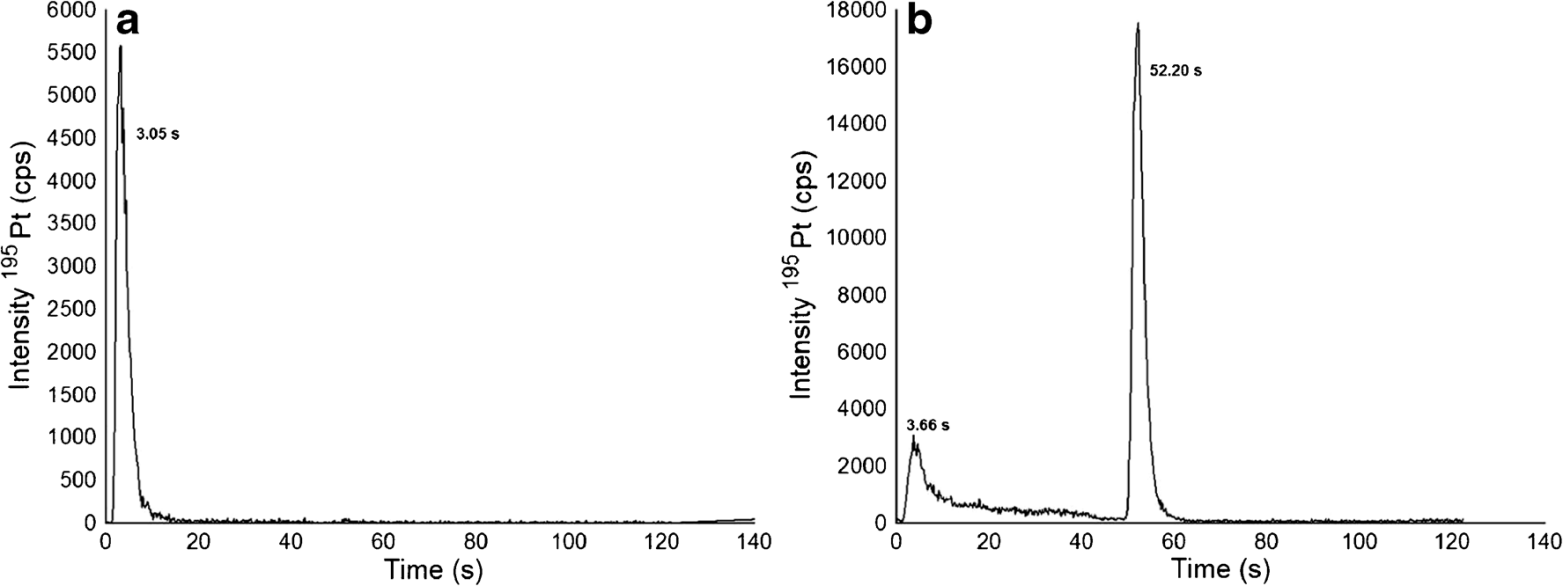
TFC chromatograms of (**A**) an incubation of KP2156 0.1 μM (20 μg/L of Pt) 1:10 in FCS during 45 min at 37 °C and diluted 1:10 in water prior to the analysis (**B**) an incubation of KP2157 0.1 μM (20 μg/L of Pt) 1:10 in FCS during 45 min at 37 °C and diluted 1:10 in water prior to the analysis. Pt was monitored at m/z 195

TFC has appeared more recently as an on-line extraction technique to study metallodrug-protein interactions, where the protein rich HMF is removed on-line by utilizing TFC. In essence, the two fractions HMF and LMF can be analyzed only considering their Pt content or alternatively, in a more sophisticated approach, the LMF is analyzed on a second analytical column [33–36]. TFC columns are available in different chemistries and dimensions [42, 43]. In this work, a silica-based fluorinated alkyl stationary phase was employed which provided a unique selectivity compared to other reversed phase chemistries [43]. TFC is a rapid analytical process compared to SEC; however, the duty cycle between samples is 9 min due to loop filling and preconditioning of the TFC column (Fig. 2). Overall, comparable throughput to UHPLC SEC could be achieved, but at the expense of higher solvent consumption necessary to reach the turbulent conditions. Figure 4 shows the TFC-ICP–MS analysis of albumin and FCS, where the protein shows virtually no retention. Figure 5 shows the results from the incubations of KP2156 and KP2157 with serum. In accordance with the fact that KP2156 shows pronounced protein binding, no retention for Pt was observed. Only the free KP2157 showed retention. The free drug (52.2 s) was retained on the column until the back flushing with MeOH was applied. TFC-ICP–MS analysis revealed two peaks for KP2157 which could be explained by the occurrence of KP2157 protein adducts and/or the generation of a hydrophilic hydrolysis product of KP2157 showing no reversed phase retention on the TFC stationary phase. The latter explanation is in accordance with UHPLC SEC-ICP–MS measurements revealing the presence of low molar mass transformation products. As can be observed in Table 6, the TFC approach provided excellent LODs in the sub nM range. As a drawback, the drug recoveries and repeatabilities were compromised compared to the UHPLC SEC-ICP–MS analysis.

**Table 6:** Analytical figures of merit of the standards and the samples

Compound	Recovery (%) *n* = 3	Precision (%) *n* = 3	LOD (nM) 3σ criterion	LOD (μg/L Pt) 3σ criterion	Absolute LOD (fg Pt) 3σ criterion
KP2157	85 ± 3	2	0.04	0.008	80
KP2156	54 ± 1	5	0.05	0.01	100
KP2156 in FCS	64 ± 2	1	0.03	0.006	60
KP2157 in FCS	90 ± 5	7	0.04	0.008	80

### Cross-validation of SEC-ICP–MS, UHPLC SEC-ICP–MS, TFC-ICP–MS, and centrifugal ultrafiltration

Finally, the discussed methods were compared regarding their suitability of quantifying the degree of protein metallodrug-adduct formation. KP2156 and KP2157 (0.1 μM, 20 μg/L of t) were incubated in fetal calf serum for 45 min at 37 °C, corresponding to a molar ratio of drug:albumin of approximately 1:6000. The incubation solutions were analyzed by conventional size-exclusion (optimal size range 0.1 kDa–10,000 kDa), (see ESM Fig. S2) by UHPLC SEC-ICP–MS (optimal size range of 1-80 kDa), (see ESM Fig. S3) by centrifugal ultrafiltration combined with flow injection analysis and finally, by TFC-ICP–MS (Fig. 5). While for the SEC- and centrifugal ultrafiltration-based methods, standard dilutions of one Pt(IV) compound served for calibration of all investigated Pt(IV) drugs and protein adducts, this species-unspecific quantification concept could not be expanded to the TFC methodology without considering the fact that the high molar mass fraction and the retained hydrophobic low molar mass fraction eluted under completely different conditions from the TFC column. Fig. S4 in the ESM shows the TFC measurements of the established calibration strategy.

Table 7 summarizes the most important findings of the cross-validation study. KP2157 was assessed in the LMF by calibration using a dilution series of the drugs. However, since Pt was < LOD in the HMF, for all methods except in TFC-ICP–MS, the bound Pt was assessed by difference to the total Pt measured by FI-ICP–MS. The opposite was true for KP2156. In this case, Pt adducts were actually quantified while the LMF was assessed as difference of the experimentally assessed Pt adduct to the total Pt. While SEC-ICP–MS and UHPLC SEC-ICP–MS were in excellent agreement for both quantification exercises, the values obtained by ultrafiltration and TFC-ICP–MS revealed inconsistencies. The former approach was compromised by poor recoveries of the free Pt(IV) compounds. Therefore, correction for recovery resulted in biased values regarding the degree of protein binding of KP2156. While with all other approaches, the LMF fraction of KP2156 was < LOD upon incubation in serum, in ultrafiltration a LMF fraction of 38% was found. TFC-ICP–MS resulted in a biased quantification in the case of KP2157. Here, a low molar mass product co-eluted with the protein fraction.

**Table 7:** Findings of the cross-validation study

Results	SEC	UHPLC SEC	Centrifugal ultrafiltration	TFC
Sample Preparation	No	No	Yes (1.25 h)	No
Time of Analysis	30 min/sample	7 min/sample	1 min/sample	9 min/sample
Solvent consumption	Medium	Medium	Low	High
Recovery (%) KP2157	93 ± 3	97 ± 3	60 ± 3^a^	90 ± 5
Recovery (%) KP2156	96 ± 1	101 ± 4	9.6 ± 2.1^a^	64 ± 2
LOD (μg/L Pt) (3σ criterion)	0.04–0.16	0.07–0.14	0.003–0.004	0.006–0.01
Assessed by calibration				
KP2157 in the LMF (%)	96 ± 2, *n* = 3	93 ± 2, *n* = 3	95 ± 4b, *n* = 9	74 ± 4, *n* = 3
KP2157 in the HMF (%)	–	–	–	38 ± 11, *n* = 3
KP2156 in the LMF (%	–	–	38 ± 6^b^, *n* = 3	–
KP2156 in the HMF (%)	102 ± 1, *n* = 3	98 ± 4, *n* = 3	–	98 ± 3, *n* = 3
Assessed by difference				
KP2157 in the LMF (%)	–	–	–	–
KP2157 in the HMF (%)	3.6 ± 1.9, *n* = 3	6.9 ± 2.5, *n* = 3	4.6 ± 4.4^b^, *n* = 9	26 ± 4, *n* = 3
KP2156 in the LMF (%)	-c	-c	–	-c
KP2156 in the HMF (%)	–	–	62 ± 6^b^, n = 3	–

^a^Corresponding to the standards of the drugs in water

^b^Using the preconditioning method with the best recoveries: 50 mM CH3COONH4, pH = 6.0 and FCS for KP2156 and KP2157 respectively (see Table 5)

^c^Below the uncertainty

## Conclusions

Different speciation methods were investigated regarding their screening capabilities towards protein binding of metallodrugs. Among the techniques, the analytical performance of the UHPLC SEC-ICP–MS suited best to perform such studies in a high-throughput manner as required in preclinical drug development without the necessity of finding dedicated analysis conditions upon investigating drugs with very different protein-binding affinity.

## Electronic supplementary material

ESM 1 (PDF 232 kb)
