# Supplementary material for: Critical assessment of different methods for quantitative measurement of metallodrug-protein associations
Source: Anal Bioanal Chem. 2018 Aug 29;410(27):7211–20. doi: 10.1007/s00216-018-1328-8 (PMC6208971; doi:10.1007/s00216-018-1328-8)
Supplement: Supplementary file 1 — (PDF 232 kb) [file 216_2018_1328_MOESM1_ESM.pdf]

Analytical and Bioanalytical Chemistry

Electronic Supplementary Material

Critical assessment of different methods for quantitative measurement of metallodrug-protein associations

Luis Galvez, Sarah Theiner, Márkó Grabarics, Christian R. Kowol, Bernhard K. Keppler, Stephan Hann, Gunda Koellensperger

Table S1 ICP-MS operation parameters and chromatographic conditions for the kinetic studies of KP2156 and KP2157 in FCS

ICP-QMS iCAP Thermo Scientific operation parameters	
Nebulizer	PFA-ST
Spray chamber	Cyclonic
Nebulizer gas flow	1.01 L/min
Aux. gas	0.99 L/min
Plasma gas	14 L/min
Reaction gas	0.370 mL/min
ICP RF Power	1550 W
m/z measured	194.97, 47.97
**Thermo Scientific Transcend system chromatographic conditions**	
HPLC Column	Acquity UPLC Protein BEH SEC, 4.6 x 150 mm, 200A, 1.7 µm, 10kDa-500kDa, Waters
Eluent	50 mM CH_3COONH_4, pH = 6.0
Flow Rate	400 µL/min
Injection Volume	2 µL
Column Temperature	37°C

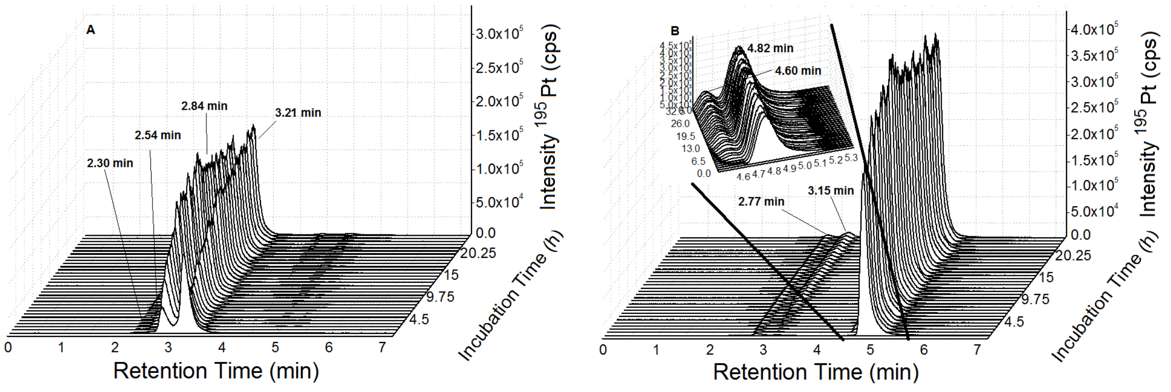

Fig. S1 Chromatographic separations with the column Acquity UPLC Protein BEH SEC, 4.6 x 150 mm, 200A, 1.7 µm, 10kDa-500kDa, of the platinum complexes (~1 µM) upon incubation in FCS during 24h at 37°C (corresponding approximately to a molar ratio of drug:albumin of ~1:600). (A) KP2156 shows a high binding to albumin (3.21, 2.84, 2.54 and 2.30 min). The free drug could not be observed. (B) KP2157 shows a lower binding to albumin (3.15 and 2.77 min). The free drug could be observed at 4.82 min. A hydrolysis product was observed in kinetics studies of the drug in water as well as in the incubation in FCS (4.60 min)

Table S2 Analytical figures of merit of the SEC and UHPLC SEC columns

Column	Compound	Retention Time (min)	N (m^{-1})[a]	H (µm)[b]
BioBasic SEC-60 A, 4.6x250 mm, 5µm, Thermo.	Methionine	12.9	17072	59
Acquity UPLC Protein BEH SEC, 4.6 x 150 mm, 125A, 1.7 µm, Waters.	Methionine	5.34	61960	16

[a] Theoretical Plate Number m^{-1}.

[b] Theoretical Plate Height µm.

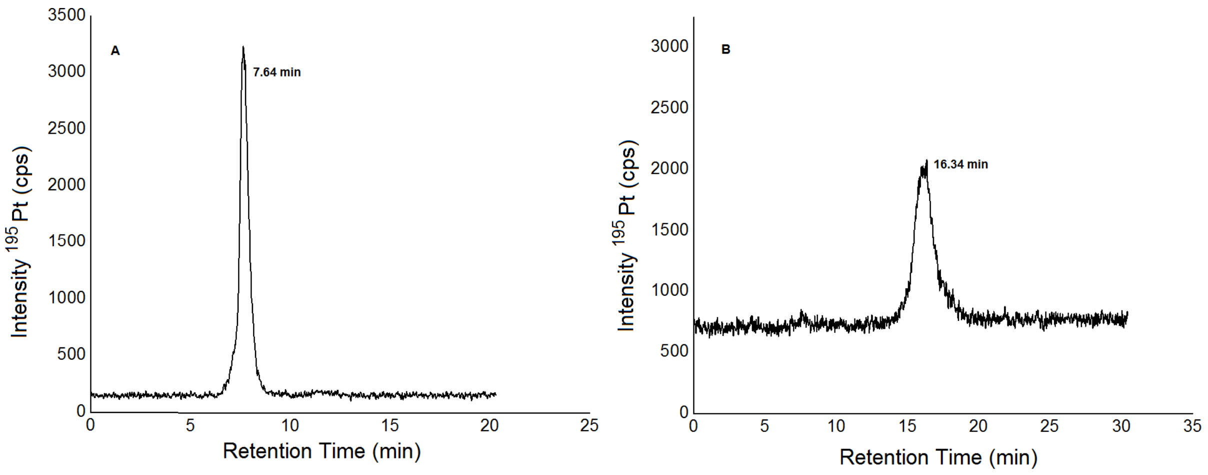

Fig. S2 SEC chromatograms (BioBasic SEC-60 A, 4.6x250 mm, 5µm) of (A) an incubation of KP2156 0.1µM (20 µg/L of Pt) 1:10 in FCS during 45 min at 37°C and diluted 1:10 in water prior to the analysis. . A high binding of this compound to albumin could be observed (7.64 min). (B) An incubation of KP2157 0.1 µM (20 µg/L of Pt) 1:10 in FCS during 45 min at 37°C and diluted 1:10 in water prior to the analysis. This compound showed no binding to albumin appearing mainly as free drug (16.34 min)

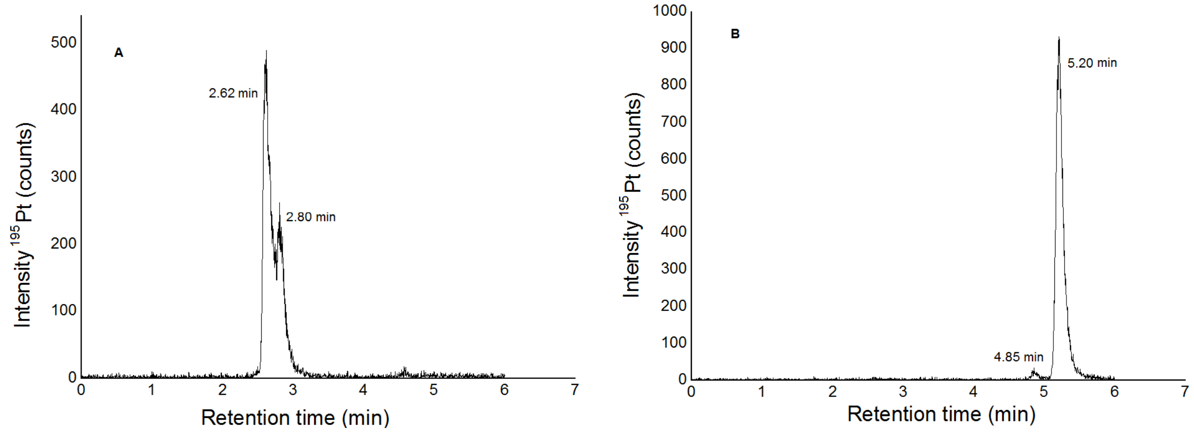

Fig. S3 UHPLC SEC chromatograms (Acquity UPLC Protein BEH SEC, 4.6 x 150 mm, 125A, 1.7 µm) of (A) an incubation of KP2156 0.1µM (20 µg/L of Pt) 1:10 in FCS during 45 min at 37°C. A high binding of this compound to the monomer and the dimer of albumin could be observed (2.62 and 2.80 min respectively) (B) an incubation of KP2157 0.1 µM (20 µg/L of Pt) 1:10 in FCS during 45 min at 37°C. This compound showed no binding to albumin appearing mainly as free drug (5.20 min). A hydrolysis product was observed in the standard of the drug in water as well as in the incubation in FCS (4.85 min)

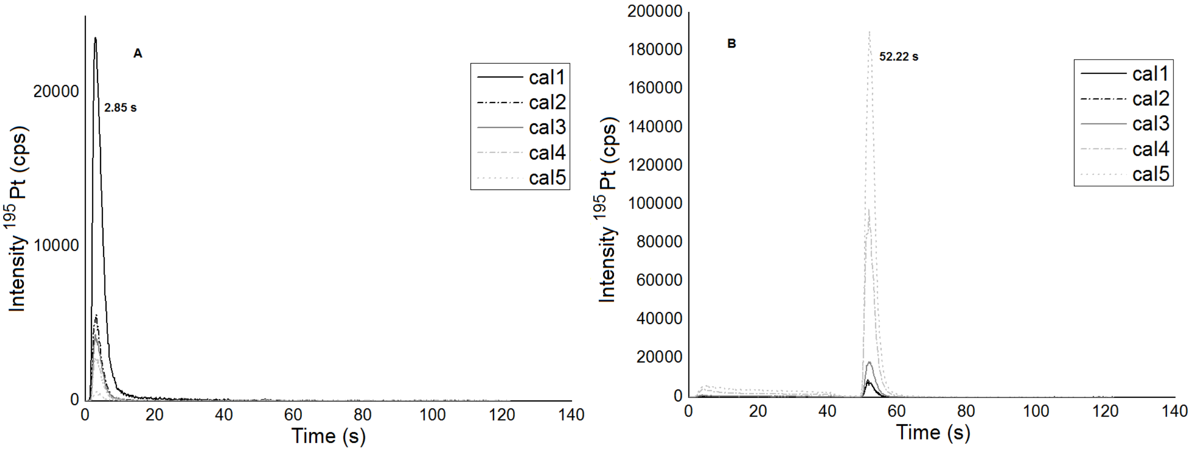

Fig. S4 TFC chromatograms (Fluoro XL 0.5x50 mm) of (A) standards of KP2156 in FCS 1:10 at different concentrations. (B) standards of KP2157 in water at different concentrations
